# Supplementary material for: The Perfect Glass Paradigm: Disordered Hyperuniform Glasses Down to Absolute Zero
Source: Sci Rep. 2016 Nov 28;6:36963. doi: 10.1038/srep36963 (PMC5125002; doi:10.1038/srep36963)
Supplement: Supplementary Information [file srep36963-s1.pdf]

The Perfect Glass Paradigm: Disordered Hyperuniform State of Matter Down to Absolute Zero Supplementary Information

G. Zhang, F. H. Stillinger, and S. Torquato

October 18, 2016

1 Three- and four-body contributions to the potential energy

To produce perfect glasses, we have used a combination of two-, three-, and four-body potentials, as specified by Sec. II in the main text. Interestingly, we also discovered that for perfect glass configurations, the three- and four-body contributions almost cancel each other. We have calculated these contributions for several inherent structures of various potential parameters using the minimum image convention. They are presented in Table 1. Because calculating the 4-body contributions in direct space is very expensive, we had to use a relatively small system size, $N = 100$.

Table 1: Three-body and four-body contributions to the potential energy for several inherent structures of $N = 100$ particles with $\gamma = 3$ and multiple α's and χ's.

α	χ	total potential energy	3-body contribution	4-body contribution
1	5.10	299.887	-97218.9	89992.4
2	5.10	693.767	-102329.5	100407.8
3	5.10	865.713	-102254.7	100242.6
6	5.10	973.172	-102436.5	100633.4
1	1.27	0.00508	-9361.75	8273.52
2	1.27	0.24845	-10357.32	10257.11

2 Energy per particle for various system sizes

To verify that the energy per particle is intensive and to quantify finite-size effects, we have calculated the energy per particle, Φ/N, for various N's for the

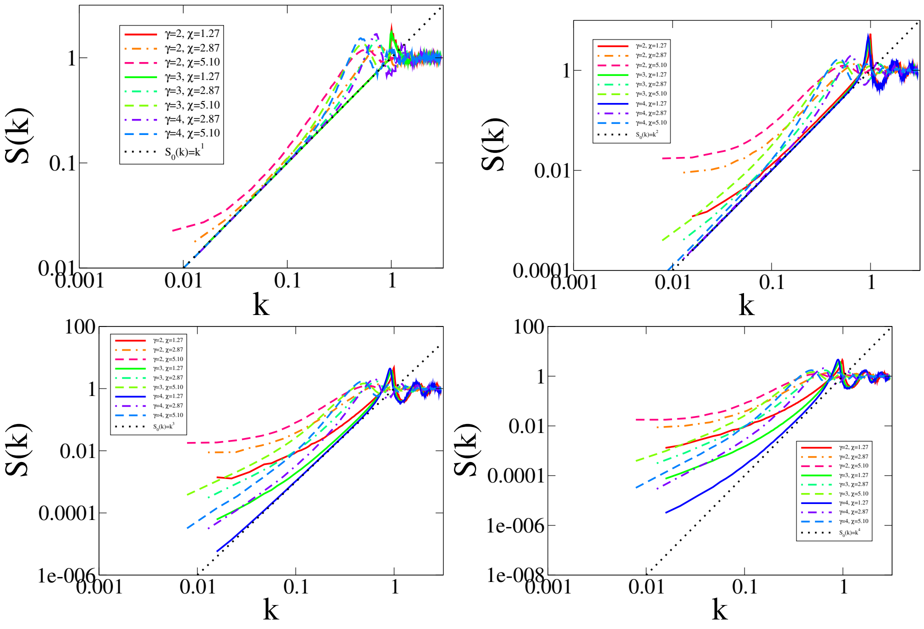

Figure 1: Structure factors of inherent structures of the perfect glass interactions in two dimensions for $\alpha = 1$ (top left), $\alpha = 2$ (top right), $\alpha = 3$ (bottom left), and $\alpha = 4$ (bottom right).

$\chi = 5.10$, $\alpha = 2$, and $\gamma = 3$ case. We generated 2,000 inherent structures of $N = 100$ particles, 100 inherent structures of $N = 2500$ particles, and 1 inherent structure of $N = 10000$ particles. The average energy per particle, $\langle \Phi/N \rangle$, is 6.99, 6.90, and 6.89, respectively, which strongly suggests the intensivity of the energy per particle in the large-system limit.

3 The conditions under which the structure factor follows the targeted shape

In the main text, we claim that the structure factor, $S(k)$, follows its target, $\mathcal{S}_0(k)$, and approaches 0 as $k \to 0$ only for $\gamma > \alpha$. Here we provide numerical evidences to support this claim.

In Fig. 1 we present $S(k)$ and $\mathcal{S}_0(k)$ for various $1 \leq \alpha \leq 4$ and $2 \leq \gamma \leq 4$. One can see that when $\gamma \leq \alpha$, $S(k)$ deviates from $\mathcal{S}_0(k)$ as k approaches zero and in some cases (e.g. the $\chi = 5.10$, $\alpha = 4$, and $\gamma = 2$ case) even appear to saturate at a positive value instead of approaching zero. However, when $\gamma > \alpha$, $S(k)$ has the same scaling as $\mathcal{S}_0(k)$ in the $k \to 0$ limit except for the $\chi = 5.10$, $\alpha = 1$, and $\gamma = 2$ case. The reason for this exception is unknown.

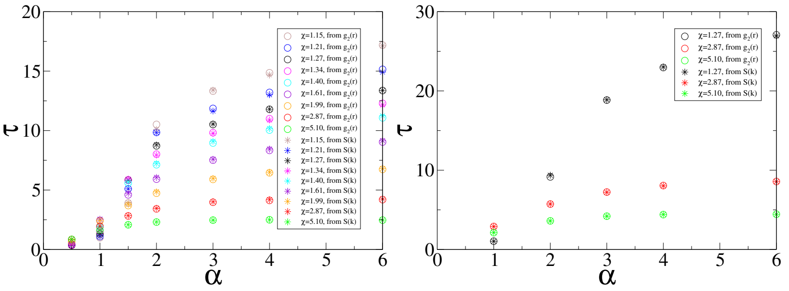

Figure 2: Order metric τ versus the exponent α for inherent structures in two (left) and three (right) dimensions for $\gamma = 3$, calculated from $g_2(r)$ or $S(k)$.

4 Elastic constants produced by non-hyperuniform targeted structure factors.

As noted in the manuscript, hyperuniform targeted structure factors generally produce much higher elastic constants than their non-hyperuniform counterparts. For $\chi = 5.10$ and $\gamma = 3$, we generated two inherent structures with the non-hyperuniform targets $\mathcal{S}_0(\mathbf{k}) = (1 + |\mathbf{k}|^2)/2$ and $\mathcal{S}_0(\mathbf{k}) = 1$. They turn out to have elastic constants $B = 0.0363$, $G = 0.0054$ and $B = 0.0174$, $G = 0.0020$, respectively. These elastic constants are to be compared, for example, to $B = 0.2025$, $G = 0.0247$, which are the ensemble-averaged elastic constants with the hyperuniform target $\mathcal{S}_0(\mathbf{k}) = |\mathbf{k}|^2$.

5 The order metric τ computed from direct space and Fourier space

As Eq. (6) of the manuscript shows, the order metric τ can be computed from either $g_2(r)$ or $S(k)$. These two approaches should find the same τ in the infinite-system-size limit for an isotropic system, but can give slightly different τ's for our finite-sized systems. In Fig. 2 we present τ calculated from both approaches. We see that τ calculated from both approaches are always very close to each other. This indicates that our calculation is accurate.

6 Visualizations of the two-, three-, and four-body contributions to the potential energy

As Appendix A in the main text shows, the perfect-glass potential can be decomposed into a sum of two-, three-, and four-body contributions. We visulize these contributions here for the $d = 2$, $\chi = 5.10$, $\alpha = 3$, and $\gamma = 2$ case. In this case the simulation box side length is $L = 800$. We have experimented with other choices of parameters and found similar results.

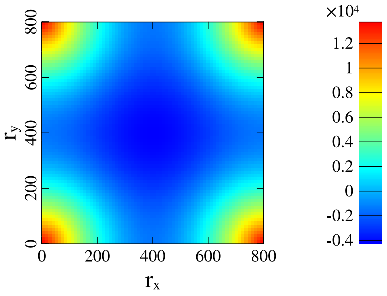

Figure 3: The two-body contribution to the perfect-glass potential, Eq. (A4) of the main text, as a function of x- and y-components of $\mathbf{r}_{lm}$

The two-body contribution, Eq. (A4) of the main text, is a function of vector $\mathbf{r}_{lm}$. We plot this function versus the x- and y-components of $\mathbf{r}_{lm}$, r_x and r_y, in Fig. 3. This contribution of the potential energy is isotropic and repulsive.

The three-body contribution, Eq. (A3) of the main text, is a function three particles' positions and is therefore much harder to visualize. We arrange the three particles as indicated in Fig. 4 and plot the three-body contribution to the potential energy for several θ values in Fig. 5. It appears that this contribution is large and positive only if all three particles are close to each other.

The four-body contribution, Eq. (A2) of the main text, is a function four particles' positions and is even harder to visulize. To visualize it we have to fix the distance between two particles to the average nearest-neighbor distance between particles. We arrange the four particles as indicated in Fig. 6 and plot the four-body contribution to the potential energy for several θ values in Fig. 7. It is interesting to note that the four-body contribution becomes very large and positive when just two particles are close to each other.

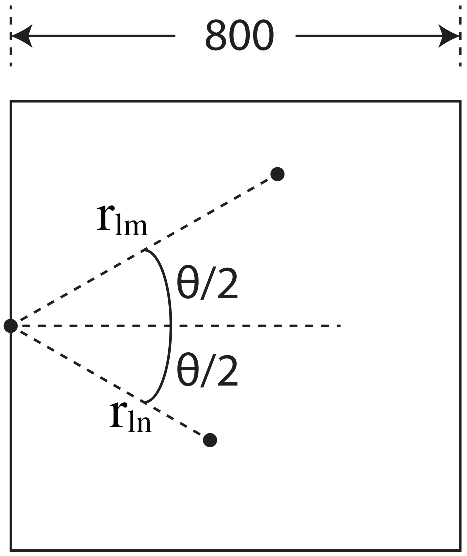

Figure 4: Arrangement of the three particles for which we plot the three-body contribution to the potential energy.

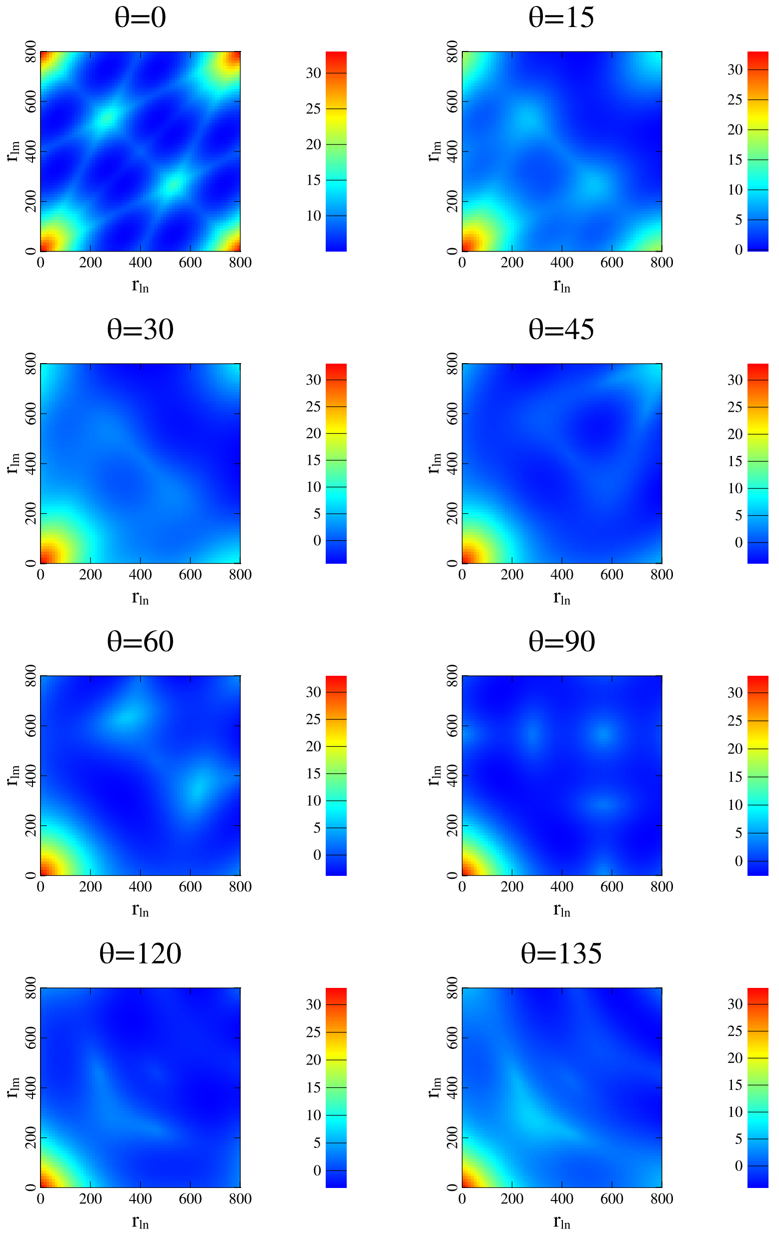

Figure 5: The three-body contribution to the perfect-glass potential, Eq. (A3) of the main text, for the three particles shown in Fig. 4, as a function of r_{lm} and r_{ln}, for $\theta = 0°$, $15°$, $30°$, $45°$, $60°$, $90°$, $120°$, and $135°$, respectively.

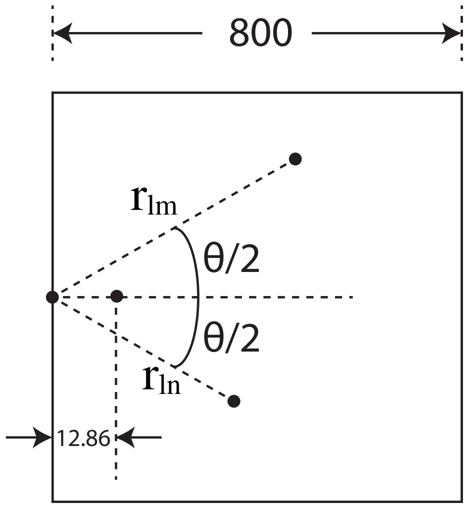

Figure 6: Arrangement of the four particles for which we plot the four-body contribution to the potential energy. The distance between two of the particles is fixed at 12.86, the average distance between a particle and its nearest neighbor in the inherent structures in the $d = 2$, $\chi = 5.10$, $\alpha = 3$, and $\gamma = 2$ case.

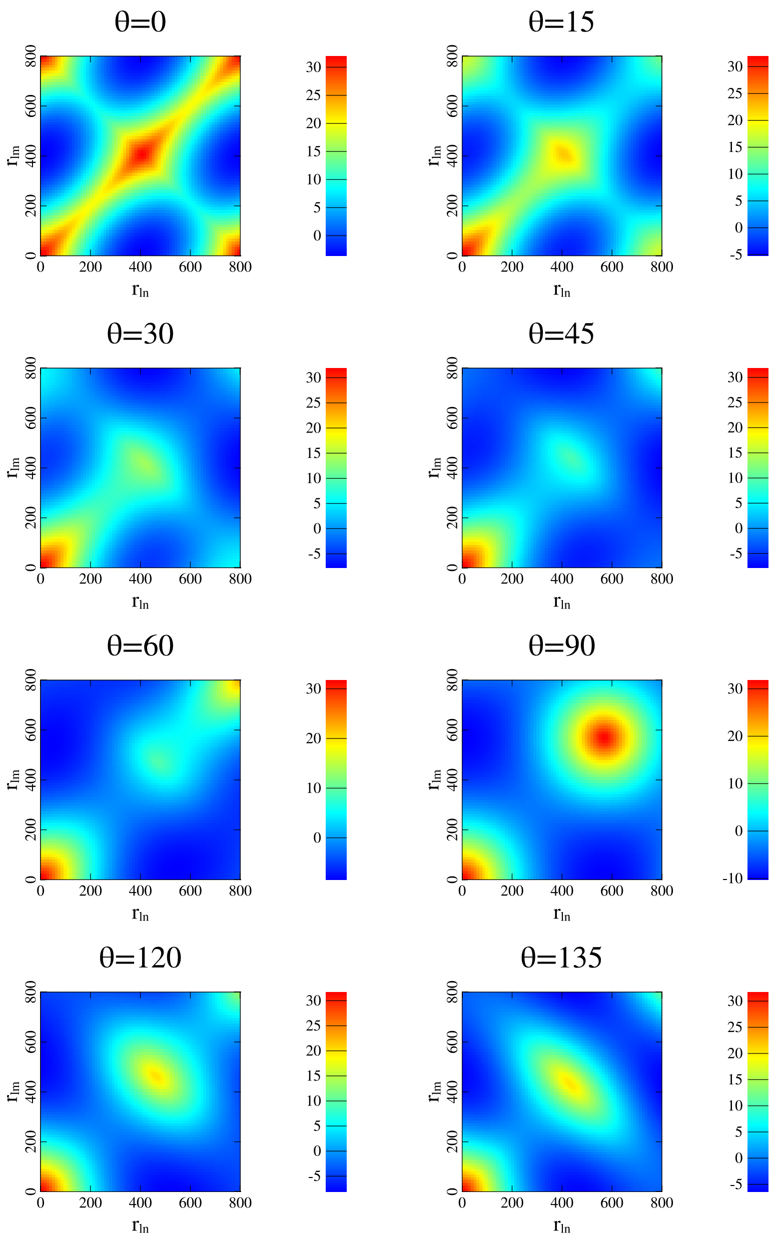

Figure 7: The four-body contribution to the perfect-glass potential, Eq. (A2) of the main text, for the four particles shown in Fig. 6, as a function of r_{lm} and r_{ln}, for $\theta = 0°$, $15°$, $30°$, $45°$, $60°$, $90°$, $120°$, and $135°$, respectively.
